# Supplementary material for: Identification of photoperiod-induced specific miRNAs in the adrenal glands of Sunite sheep (Ovis aries)
Source: Front Vet Sci. 2022 Jul 22;9:888207. doi: 10.3389/fvets.2022.888207 (PMC9354845; doi:10.3389/fvets.2022.888207)
Supplement: Supplementary file 1 [file Data_Sheet_1.ZIP › Supplementary file/miRDeep2 file.zip!/miRDeep2 file/Know_Structure/LP42A2/oar-mir-19b.pdf]

miRBase precursor : oar-mir-19b
Total read count : 2480
oar-miR-19b read count : 2473
remaining reads : 7

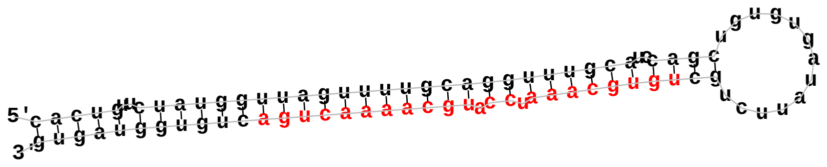

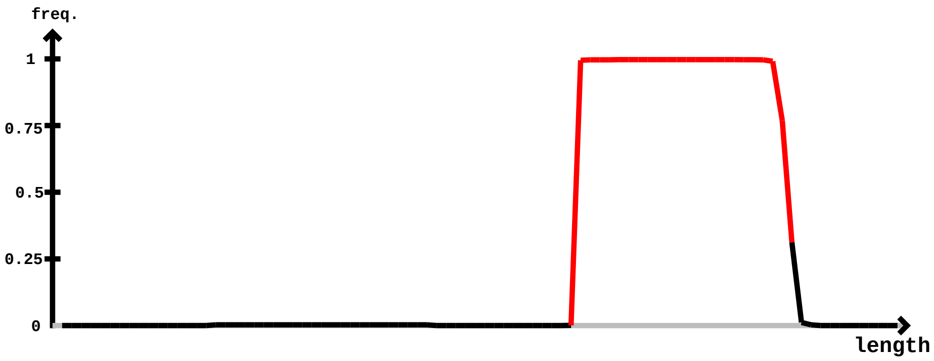

oar-miR-19b

```
5'- cacuguucuaugguuaguuuugcagguuugcauccagcugugugauauucugcugugcaaauccaugcaaaacugacugugguagug   -3'    exp
    (((((..(((((((((((((((((((((((((..((((.............)))))))))).)).))))))))))))))))))))))    reads      mm         sample
    ...............aguuuugcagguuugcauccagc.................................................    7          0          seq
    ....................................................cugugcaaauccaugcaaaacug............    1          0          seq
    ....................................................cugugcaaauccaugcaaaacuga...........    1          0          seq
    .....................................................ugugcaaauccaugcaa.................    1          0          seq
    .....................................................ugugcaaauccaugcaaaa...............    1          0          seq
    .....................................................ugugcaaaucUaugcaaaac..............    1          1          seq
    .....................................................ugugcaaauccaugcaaaac..............    12         0          seq
    .....................................................ugugcaaaucUaugcaaaacu.............    88         1          seq
    .....................................................ugugcaaauccaugcaaaacu.............    465        0          seq
    .....................................................ugugcaaaucUaugcaaaacug............    88         1          seq
    .....................................................ugugcaaauccaugcaaaacug............    1037       0          seq
    .....................................................ugugcaaauccaugcaaaacuga...........    625        0          seq
    .....................................................ugugcaaaucUaugcaaaacuga...........    118        1          seq
    .....................................................ugugcaaauccaugcaaaacugaU..........    20         1          seq
    .....................................................ugugcaaauccaugcaaaacugac..........    2          0          seq
    .....................................................ugugcaaauccaugcaaaacugaUu.........    7          1          seq
    ......................................................gugcaaauccaugcaaaacug............    3          0          seq
    ......................................................gugcaaauccaugcaaaacuga...........    1          0          seq
    .........................................................caaauccaugcaaaacug............    2          0          seq
```
